# The vertebro-subclavian artery angle modulates hemodynamic mechanisms of atherosclerosis in vertebral artery origin: a combined clinical and computational fluid dynamics study

**DOI:** 10.3389/fbioe.2026.1775214

**Published:** 2026-04-21

**Authors:** Ligang Chen, Bin Hou, Chuangzhong Li, Yuan Cao, Xiaogang Wang, Guobiao Liang

**Affiliations:** 1 Department of Neurosurgery, General Hospital of the Northern Theatre Command, Shenyang, China; 2 Department of Neurosurgery, Affiliated Hospital of Shandong University of Traditional Chinese Medicine, Jinan, China; 3 Department of Neurosurgery, Affiliated Hospital of Liaoning University of Traditional Chinese Medicine, Shenyang, China

**Keywords:** atherosclerosis, computational fluid dynamics, haemodynamics, time averaged wallshear stress, vertebral artery origin

## Abstract

**Objective:**

By dictating local hemodynamics, vascular geometry played a critical role in atherosclerosis (AS). The objective of this research was to clarify how the vertebro-subclavian artery (VA-SCA) angle affected local blood flow patterns in the pathophysiology of AS at the vertebral artery origin (VAO).

**Methods:**

This research integrated clinical retrospective analysis with computational fluid dynamics (CFD) simulation. The clinical component examined 364 VAs and classified them into Plaque/Stenosis-free and Plaque/Stenosis groups based on DSA images. Baseline data and morphological parameters (VAO length, tortuosity, VA-SCA angles, and lateralisation) were analysed and compared between the two groups. For the CFD component, idealised geometric models with variable VA-SCA angles were established, and a comprehensive investigation was conducted to elucidate the effects of angle variations on the medial and lateral walls of the VAO region with respect to time-averaged wall shear stress (TAWSS), oscillatory shear index (OSI), and hemodynamic flow field characteristics. Furthermore, these hemodynamic trends derived from parametric modelling have been validated by patient-specific data.

**Results:**

In this retrospective clinical study, multivariate regression analysis revealed that a larger VA-SCA angle (*OR* = 1.84, *95% CI*: 1.46–2.33, *P* < 0.001) and severe curvature (*OR* = 2.35, *95% CI:* 1.21–4.56, *P* = 0.011) were independent risk factors for AS at the VAO. CFD simulations indicated that across all angle configurations, the lateral VAO wall consistently showed lower TAWSS and higher OSI than the medial wall, with vortex formation observed in all models. With an increase in the VA-SCA angle, the lateral wall TAWSS initially decreased before increasing again, attaining its minimum at 50°, whereas the OSI displayed an opposite trend, peaking at 70°. Analysis of patient-specific data further confirmed that, as the VA-SCA angle increases, TAWSS initially decreases, then increases. At the same time, OSI shows an initial increase followed by a decline.

**Conclusion:**

The VA-SCA angle may influence the pathogenesis of AS at the VAO through hemodynamic factors and could serve as a new imaging biomarker to assess hemodynamics in this context.

## 1 Introduction

Atherosclerosis-related disorders have been the predominant cause of mortality globally, accounting for almost one-third of all fatalities from cardiovascular and cerebrovascular incidents, thereby imposing a considerable public health burden (Roth et al., 2020; Chen H. et al., 2025; GBD 2019 Stroke Collaborators, 2021). Following the carotid bifurcation, the vertebral artery origin (VAO), commonly referred to as the V1 segment, has the second-highest prevalence of atherosclerosis (AS) (Krajíčková et al., 2024; Qtaish et al., 2024; Chen H. et al., 2025).

Emerging investigations have underscored hemodynamic factors as a novel research paradigm for elucidating the pathogenesis of atherosclerosis (Ekmejian et al., 2024; Vuong et al., 2024; Malek et al., 2025; Yang et al., 2025; Moghadasi et al., 2026). Blood flow exerts various forces on the vessel wall, including circumferential forces, radial forces, and wall shear stress (WSS) (Grégory, 2021). WSS was the most thoroughly investigated parameter in current research, defined as the frictional force between blood flow and endothelial cells (Cho et al., 2024). Normal or slightly elevated WSS (1–7 Pa) had anti-atherosclerotic effects by promoting the ordered alignment of endothelial cells in the direction of blood flow, downregulating inflammatory agents, and enhancing nitric oxide (NO) release via the upregulation of endothelial nitric oxide synthase (eNOS), which facilitated vasodilation and inhibited platelet adhesion and aggregation (Qian et al., 2020). A low WSS (less than 1 Pa) was a significant factor in the progression of AS, as it contributed to endothelial dysfunction, lipid accumulation, and increased inflammatory responses (Ekmejian et al., 2024). Researchers have introduced the oscillatory shear index (OSI) to evaluate the extent of directional variation in WSS throughout a cardiac cycle. The OSI range was from 0 to 0.5, with higher values indicating increased volatility in blood flow direction (Cho et al., 2024).

Nowadays, advances in computational fluid dynamics (CFD) have enabled researchers to numerically quantify the local hemodynamic environment in AS-prone regions, thereby elucidating the mechanisms by which morphological factors drive AS (Vuong et al., 2024). Research on carotid and coronary bifurcations indicated that increased bifurcation angles were associated with AS stenosis, which was supported by hemodynamic analysis via CFD simulations (Sun and Chaichana, 2017). However, research on the correlation between stenosis and VAO morphology remains substantially underdeveloped. Although many studies have suggested that morphological parameters, including vertebral artery (VA) asymmetry and VAO curvature, are correlated with the severity of stenosis (Zhu et al., 2016), the parameters discussed in these studies are not sufficiently systematic or comprehensive. Additionally, there was a lack of studies on VAO, which integrated CFD with clinical studies to thoroughly examine the underlying pathogenesis mechanisms from a hemodynamics standpoint.

An integrated methodology of clinical retrospective analysis, CFD simulation, and validation by patients’ data was implemented in this investigation. Firstly, we conducted a statistical analysis of the morphological characteristics of the VAO using the data from computed tomography angiography (CTA) and identified risk factors affecting the degree of AS. Second, we utilised CFD to create idealised bifurcation models of the vertebro-subclavian artery (VA-SCA) to examine the effects of bifurcation angle modifications on key hemodynamic parameters in the area of VAO. Finally, the observed trends were validated using real-world patient data. The objective of this investigation was to enhance understanding of the pathogenesis of AS at the VAO through morphological and fluid dynamic analyses, thereby providing theoretical support for the early identification of anatomical configurations that were at high risk of AS.

## 2 Materials and methods

### 2.1 Study design

This research consisted of two components: the clinical retrospective study and CFD simulations. A retrospective clinical study enrolled 182 patients with cerebral atherosclerosis admitted to the Department of Neurosurgery at Northern Theatre General Hospital, following established inclusion and exclusion criteria, with baseline data collected accordingly. Morphological parameters were derived from the CTA images of patients’ heads and necks, and the Plaque/Stenosis at the VAO was assessed using digital subtraction angiography (DSA) results. The CFD simulation section comprised the development of models with different VA-SCA angles to conduct CFD simulations on the VAO, thus acquiring pertinent fluid dynamics parameters. The ethics committee of the hospital approved this study.

### 2.2 Clinical retrospective study

#### 2.2.1 The criteria for patients

Inclusion criteria: (1) comprehensive general information available from our centre; (2) complete CTA and DSA imaging data obtained from our hospital; (3) age range of 50–80 years.

Exclusion criteria include: (1) abnormal origin of the vertebral artery; (2) prior vertebral artery stenting or stripping surgery; (3) narrowing, occlusion, aneurysm, vascular malformation, or developmental abnormalities in VA of segments V2-V4, basilar artery, or posterior cerebral artery; (4) concurrent subclavian artery stenosis or occlusion; (5) variant vertebral artery entry into the intervertebral foramen; (6) concurrent autoimmune diseases such as arteritis or systemic lupus erythematosus; (7) history of radiation or chemotherapy.

#### 2.2.2 CTA

This study employed a Philips CT scanner with the following parameters: tube voltage, 120 kV; tube current, 30 mA; matrix, 512 × 358; reconstruction slice thickness and reconstruction interval, both set at 0.60 mm; and a scanning range extending from the aortic arch to the cranial vault. Using a high-pressure syringe, inject 50 mL of iopamidol contrast agent (iodine ion concentration 32g/100mL, Jiangsu Hengrui Medicine Co., Ltd.) at a rate of 10 mL/s. Subsequently, inject 50 mL of 0.9% sodium chloride injection at the same rate to obtain the raw CT images. Then, reconstruct and measure the original image in the AQi system to obtain VAO morphological parameters.

#### 2.2.3 DSA

This study utilised a dual-C-arm Siemens DSA device. Firstly, disinfect both femoral arteries. After successful femoral artery puncture using the modified Seldinger approach, exchange the J-wire and insert a 5F vascular sheath. A 5F single-curve angiographic catheter was advanced through the short sheath using a lizard wire. The lizard wire was withdrawn into the catheter after passing the aortic arch. The single-curve catheter was then retracted and positioned within the subclavian artery. The catheter was adjusted to the proximal vertebral artery origin, and the lizard wire was withdrawn. Adjust the positioning plate to the Towne position. Using a high-pressure injector, administer 15 mL of iopromide contrast agent (iodine ion concentration 100 mL:32 g, Jiangsu Hengrui Medicine Co., Ltd.) at a rate of 6 mL/s. Obtain DSA images to evaluate the Plaque/Stenosis at the VAO.

#### 2.2.4 Data collection

Collect baseline data for study subjects, including age, gender, and underlying medical conditions (diabetes, coronary heart disease, hypertension); as well as fasting blood glucose, triglyceride (TG), low-density lipoprotein (LDL), and homocysteine (Hcy) levels.

**FIGURE 1:**
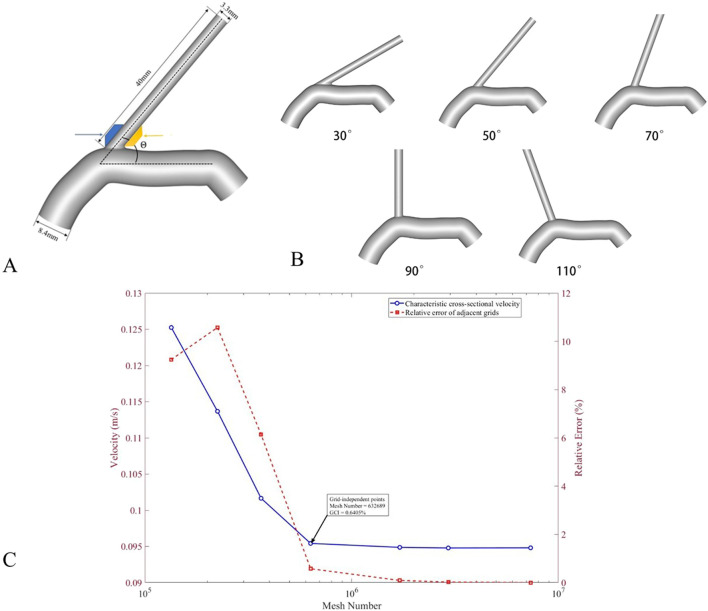
**(A)** Key geometric specifications of the idealised VA-SCA bifurcation model include a VA diameter of 3.3 mm, an SCA diameter of 8.4 mm, a VAO length of 40 mm, and the VA-SCA angle (θ). The blue and yellow areas denote the lateral and medial walls, respectively. **(B)** A series of generated models with VA-SCA angles of 30°, 50°, 70°, 90°, and 110°. **(C)** Mesh independence verification for CFD simulations. Seven mesh models with progressively increasing refinement (element counts ranging from 134,091 to 7,336,488; corresponding mesh sizes from 0.6 mm to 0.1 mm) were generated and tested. The average velocity at a characteristic cross-section sensitive to flow features was monitored as the convergence indicator. Relative errors between adjacent mesh levels decreased with refinement, stabilising below 1% when the mesh count exceeded 632,689 (0.3 mm mesh size), indicating that further mesh refinement has a negligible impact on the velocity results. To further quantify discretisation error, the Grid Convergence Index (GCI) method was applied to three representative mesh levels (0.3 mm, 0.2 mm, and 0.1 mm), yielding a GCI value of 0.6405% for the finest mesh. Based on these results, the 0.3 mm mesh (approximately 630,000 elements) was adopted for all subsequent simulations, balancing computational accuracy and resource efficiency.

Using head and neck CTA to measure morphological parameters of the VAO (Figure 1A), including VAO length, VA-SCA angle, lateralisation, and degree of curvature. VAO length was defined as the distance from the origin of the VA at the SCA to the C6 transverse foramen; the VA-SCA angle was defined as the angle formed between the origin of the VA and the distal end of the SCA. This study employed the VAO morphological classification proposed by Zhou et al. (2011), which included Type I, Type C, reverse Type C, Type S, and reverse Type S. Based on these classifications, curvature was categorised as: straight type (Type I), mildly curved type (Type C, reverse Type C), and severely curved type (Type S, reverse Type S). Vertebral arteries with plaques or stenosis identified by DSA imaging were classified into the Plaque/Stenosis group; normal vertebral arteries were classified into the Plaque/Stenosis-free group. Both CTA and DSA data were independently measured by two physicians, with the mean value taken to ensure accuracy.

#### 2.2.5 Statistical analysis

Data were processed using SPSS statistical software (version 26.0; IBM Corporation, Somers, NY, USA). The Kolmogorov-Smirnov test was employed to assess the normality of measurement data. For normally distributed quantitative data, results were expressed as $\bar{x}$ *±s*, and *t*-tests were used to compare intergroup differences. For non-normally distributed quantitative data, results were presented as *M* (*P*_25_, *P*_75_), and Wilcoxon rank-sum tests were used to compare intergroup differences. For categorical data such as VAO morphology and side, results were expressed as percentages (%), and chi-square tests were used to compare intergroup differences. With vertebral artery atherosclerosis/stenosis as the dependent variable, include intergroup-differentiated independent variables in a multivariate logistic regression analysis. Define *P* < 0.05 as indicating statistically significant differences.

### 2.3 Computational fluid dynamics

#### 2.3.1 Model construction

In the SolidWorks 2024 software (SolidWorks Corporation, France), process the left VA-SCA bifurcation model by closing the openings of the internal thoracic artery, converting them into smooth surfaces, and retaining only the SCA and VA (Figure 1A). According to the references, the internal diameter of the SCA was 8.4 mm (Yuan, 2016) and the VA was 3.3 mm (Li et al., 2019), with a wall thickness of 0.5 mm. Set the VA-SCA angle to 30°, 50°, 70°, 90°, and 110° to establish geometric models (Figure 1B). Angle control accuracy reached ±0.5°, and the models were exported in STEP format.

#### 2.3.2 Construction and independence verification of mesh models

Prior to conducting formal numerical simulations, this study performed a systematic mesh-independence verification for the computational domain to eliminate the influence of mesh discretisation errors on the calculation results and to determine a reasonable mesh scheme that balances computational accuracy and resource consumption. The verification was carried out based on seven sets of meshes with different refinement levels. The geometric model in STEP format was imported into HyperMesh 2022 (Altair Engineering Inc., USA) for meshing. The resulting mesh counts, from coarse to fine, were 134,091; 224,354; 364,527; 632,689; 1,708,459; 2,932,468; and 7,336,488. These meshes were saved in CAS format and imported into Ansys Fluent 2023 R2 (ANSYS, Inc., USA). The corresponding characteristic mesh sizes were 0.6 mm, 0.5 mm, 0.4 mm, 0.3 mm, 0.2 mm, 0.15 mm, and 0.1 mm, respectively. The average velocity on a characteristic cross-section sensitive to flow features was selected as the monitoring parameter to compare the simulation results obtained from the different mesh systems in Fluent.

The relative error between two adjacent mesh levels (with the coarser mesh as the reference) is defined as follows, Equation 1:

$$\varepsilon =\left|\frac{v_{\text{coarse}}-v_{\text{fine}}}{v_{\text{coarse}}}\right|\times 100\%$$ (1)

The results show that the relative error decreases as the mesh is refined: when the mesh count increases from 134,091 to 224,354, the relative error is 9.24%; from 224,354 to 364,527, it is 10.57%; from 364,527 to 632,689, it drops to 6.14%; from 632,689 to 1,708,459, it further decreases to 0.57%; and with further refinement to 2,932,468 and 7,336,488, the relative errors are only 0.088% and 0.021%, respectively. It is evident that, starting from the mesh count of 632,689, the relative error between adjacent mesh levels stabilises below 1%, indicating that further mesh refinement has a negligible impact on the velocity results.

To further quantify the discretisation error, the Grid Convergence Index (GCI) method was applied to three mesh levels (0.3 mm, 0.2 mm, and 0.1 mm). The calculated GCI value for the finest mesh is 0.6405%, demonstrating that the discretisation error remains at a low level.

Considering both computational accuracy and resource consumption, all subsequent simulations in this study adopted the mesh topology and node arrangement corresponding to the 0.3 mm mesh size for discretising models with various branch angles (Figure 1C).

#### 2.3.3 Parameter settings

Ensure the vessel wall is rigid and non-slip by setting its density to a blood density of 1060 kg/m^3^. Blood was treated as an incompressible non-Newtonian fluid with a viscosity coefficient set according to the Casson viscosity model (Sean et al., 2023). Based on the Reynolds number formula, the Reynolds number was <2300; thus, the calculation employed laminar flow. The coupling algorithm utilised the SIMPLE algorithm.

Blood was a typical non-Newtonian fluid, exhibiting characteristic non-linear behaviour during flow within the body. When shear stress exceeded the critical threshold, blood exhibited shear thickening in its dynamic viscosity, which differed from the assumption in most studies that blood was a Newtonian fluid. This project employed the Casson rheological model to develop user-defined functions (UDFs) for mathematical characterisation, which was defined as follows, Equation 2:

$$\mu \left(\dot{\gamma }\right)=\frac{\mu_{\infty }^{2}}{\dot{\gamma }}+\frac{2\mu_{\infty }N_{\infty }}{\sqrt{\dot{\gamma }}}+N_{\infty }^{2}$$ (2)

$N_{\infty }$ described the non-linear increase in viscosity resulting from an increase in hematocrit (Hct). ${\left(1-Hct\right)}^{-\frac{1}{4}}$ represented the empirical correction factor. It was defined as follows, Equation 3:

$$N_{\infty }=\sqrt{\mu_{p}{\left(1-Hct\right)}^{-\frac{1}{4}}}$$ (3)

$\mu_{\infty }$ represented the asymptotic viscosity of blood at extremely high shear rates and was positively correlated with the square root of red blood cell concentration, reflecting the ultimate state of shear thinning. It was defined as follows, Equation 4:

$$\mu_{\infty }=\sqrt{\frac{5}{8}Hct}$$ (4)

Plasma viscosity $\mu_{p}$ was 0.00145 Pa·s ; Hct was 0.4 g/dL.

A three-dimensional blood flow numerical model was constructed using a dynamic fluid domain discretisation method. The inlet boundary conditions employed a pulsatile velocity profile with a peak flow velocity of 16 cm/s in its fundamental frequency component. The waveform configuration followed the theoretical solution of Womersley’s arterial flow theory. The Reynolds number range of Re = 500–1200 characterised laminar-transitional flow regimes. The outlet boundary was set to a standardised atmospheric pressure reference (102.3 kPa gauge pressure), with a pulsatile pressure component superimposed at a phase difference of π/2 to simulate the characteristics of peripheral vascular impedance.

The inlet pulsation velocity profile was defined as follows, Equation 5:

$$U\left(t\right)=\frac{Q\left(t\right)}{A}$$ (5)

In the above formula, U(t) represented the pulsation velocity profile (cm/s), Q(t) denoted the pulsation volumetric flow rate (cm^3^/s), and A was the inlet cross-sectional area (cm^2^).

Additionally, the flow-pressure relationship was represented by a fourth-order Fourier series, which was defined as follows, Equation 6 and Equation 7:

$$Q\left(t\right)=\bar{Q}+\sum_{n=1}^{4}a_{n}^{Q}\cos\left(n\omega t\right)+\beta_{n}^{Q}\sin\left(n\omega t\right)$$ (6)

$$P\left(t\right)=\bar{P}+\sum_{n=1}^{4}a_{n}^{P}\cos\left(n\omega t\right)+\beta_{n}^{P}\sin\left(n\omega t\right)$$ (7)

The parameters in the above formula were as shown in Table 1.

**TABLE 1:** Wave equation parameter table.

Artery	n	$a_{n}^{Q}$	$\beta_{n}^{Q}$	$a_{n}^{P}$	$\beta_{n}^{P}$
$\bar{Q}=2.6483\;cm^{3}/s$	1	0.1007	0.0764	−3.3107	−2.2932
$\bar{P}=11328.663\;Pa$	2	−0.0034	−0.0092	−9.8639	8.0487
​	3	0.0294	0.0337	3.0278	3.8009
​	4	0.0195	−0.0129	2.2476	−3.2564

Referencing the average resting heart rate of healthy adults (75 beats per minute), the cardiac cycle period T was set to 0.8 s. The wall surface was defined with a no-slip boundary condition (v = 0), and the WSS threshold was set to 5 Pa to prevent computational errors.

#### 2.3.4 CFD parameter

TAWSS: It referred to the average shear stress exerted by a fluid on a wall surface during periodic flow. It was a scalar parameter obtained by time-averaging the instantaneous WSS over a complete cycle.

TAWSS was defined as the root mean square value of the time integral of the WSS vector during a cardiac cycle, which was defined as follows, Equation 8:

$$\text{TAWSS}=\frac{1}{T}\int_{0}^{T}\left|\text{WSS}\left(t\right)\right|\;dt$$ (8)

In the above formula, $\text{WSS}\left(t\right)$ represented the instantaneous shear stress vector on the wall surface; T=0.8 s denoted the complete cardiac cycle. The shear stress direction was calculated from the normal component of the velocity gradient tensor, which was defined as follows, Equation 9:

$$\text{WSS}=\mu \left(\dot{\gamma }\right)\cdot \left(\nabla v+{\left(\nabla v\right)}^{T}\right)\cdot n$$ (9)

In the above formula, n represented the wall normal vector, v denoted the velocity of the wall mesh element, and viscosity μ was updated in real time via the Casson model UDFs.

OSI: it characterised the pulsation intensity of shear stress direction and quantified the mechanical stimulation of fluid oscillations on vascular endothelium, which was defined as follows, Equation 10:

$$\text{OSI}=\frac{1}{2}\left(1-\frac{\left|\int_{0}^{T}\text{WSS}\left(t\right)\;dt\right|}{\int_{0}^{T}\left|\text{WSS}\left(t\right)\right|\;dt}\right)$$ (10)

The OSI value range was (0, 0.5). The OSI >0.2 indicated significant oscillatory flow; the OSI <0.2 indicated a quasi-steady shear environment (Ekmejian et al., 2024).

The above definitions were written as a UDF and loaded into the model imported into Ansys Fluent 2023 R2. The cardiac cycle period T was set to 0.8 s, and two cardiac cycles were simulated. After the simulation, the TAWSS and OSI values were extracted from the second cardiac cycle.

#### 2.3.5 Parameter calculation

Employing the eigenvalue threshold segmented sampling method, quantified the distribution of biomechanical parameters in the VA-SCA zone. Establish a coordinate system along the vascular axis within Ansys Fluent 2023 R2 software (with the Z-axis corresponding to the primary blood flow direction). Identify two typical physiological feature zones within the OSI characteristic spectrum: the critical oscillation zone on the lateral wall and the steady-state shear zone on the medial wall. Subsequently, the surface parameter integral method was applied to obtain the average TAWSS and OSI values for the two characteristic regions (medial wall and lateral wall), respectively.

## 3 Result

### 3.1 Baseline data

The mean age of the 182 patients (364 VAs) in this study was 64.74 years. In contrast to the Plaque/Stenosis group, which had 158 VAs (0.43), the Plaque/Stenosis-free group had 206 VAs (0.57). No statistically significant differences were found between the two groups for other indicators (*P* > 0.05). Baseline data were presented in Table 2.

**TABLE 2:** Patient baseline characteristics.

Characteristics	Plaque/Stenosis-free group	Plaque/Stenosis group	*P*-value
Age (y, $\bar{x}$ *±s*)	64.37 ± 6.86	65.23 ± 6.90	0.238
Gender, male [n (%)]	161 (0.78)	129 (0.82)	0.433
Hypertension [n (%)]	144 (0.69)	120 (0.76)	0.200
Diabetesmellitus [n (%)]	66 (0.32)	60 (0.38)	0.251
Coronary atherosclerotic heart disease [n (%)]	47 (0.23)	29 (0.18)	0.299
Fasting blood glucose [mmol/L, *M(P25,P75)*]	5.42 (4.84, 6.38)	5.55 (5.04, 6.81)	0.534
LDL [mmol/L, *M(P25,P75)*]	1.84 (1.59, 2.41)	1.93 (1.67, 2.51)	0.321
Hcy [mmol/L, *M(P25,P75)*]	12.73 (10.42, 16.02)	12.38 (10.08, 16.38)	0.877
TG [mmol/L, *M(P25,P75)*]	1.27 (0.94, 1.84)	1.24 (0.94, 1.84)	0.989

LDL, low-density lipoprotein; TG, triglyceride; Hcy, homocysteine.

### 3.2 Correlation analysis between VAO morphology and stenosis severity

When morphological data were examined across groups, the Plaque/Stenosis group’s VA-SCA angle was substantially larger than that of the control group (79.90° vs. 65.50°, *P* < 0.05), and a significant difference in vascular tortuosity was observed between groups (P < 0.05). No intergroup differences were observed in VAO length or lateralisation (Table 3). An increased VA-SCA angle (*OR* = 1.84, 95% *CI*: 1.46–2.33, *P* < 0.001) and severe curvature (*OR* = 2.35, 95% *CI*: 1.21–4.56, P = 0.011) were also found to be independent risk variables for AS at VAO by multivariate logistic regression analysis (Table 3).

**TABLE 3:** Relationship between the morphology of VAO and stenosis.

Characteristics	Plaque/Stenosis-free group	Plaque/Stenosis group	*P* value*	Multivariate logistic regression		
				*OR*	*95%CI*	*P* value*
Length (mm)	39.33 ± 8.48	39.29 ± 9.09	0.968	​	​	​
Lateralization	​	​	0.468	​	​	​
Left	100	82	​	​	​	​
Right	106	76	​	​	​	​
VA-SCA Angle (°)	65.50 (47.60, 79.95)	79.90 (61.85, 96.28)	<0.001	1.84	(1.46, 2.33)	<0.001
Tortuosity	​	​	0.014	​	​	​
Straight type	80	44	​	​	​	​
Mildly curved type	102	74	​	1.34	(0.82, 2.19)	0.237
Severely curved type	24	40	​	2.35	(1.21, 4.56)	0.011

* *P* < 0.05 indicates statistical significance.

OR, odds ratio; CI, confidence interval.

### 3.3 VA-SCA hemodynamic analysis

**FIGURE 2:**
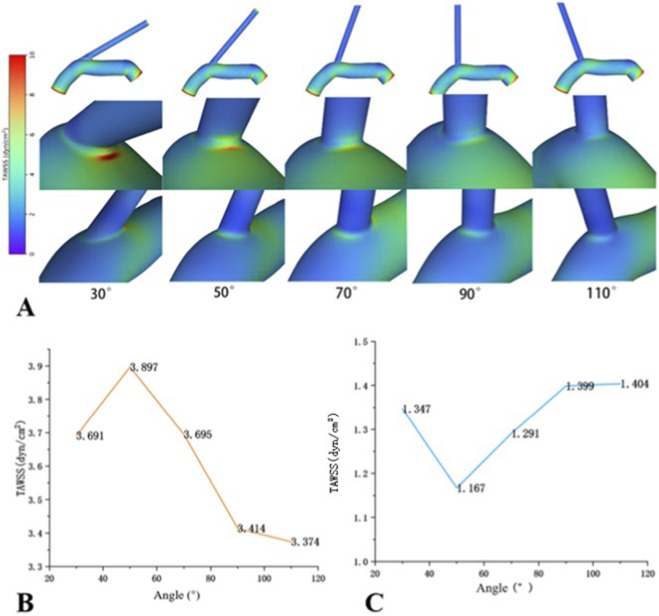
TAWSS distribution of VA-SCA based on the models. **(A)** At the same angle, the TAWSS _lateral_ is consistently lower than the TAWSS _medial_. **(B)** TAWSS _medial_ across the bifurcations. As the angle increases, the TAWSS _medial_ first increases and then decreases, reaching its maximum of 3.597dyn/cm2 at 50°. **(C)** TAWSS _lateral_ of the VA-SCA in 30°–110° bifurcations, which exhibited an opposing trend, reaching a minimum of 1.167 dyn/cm2 at 50°.

**FIGURE 3:**
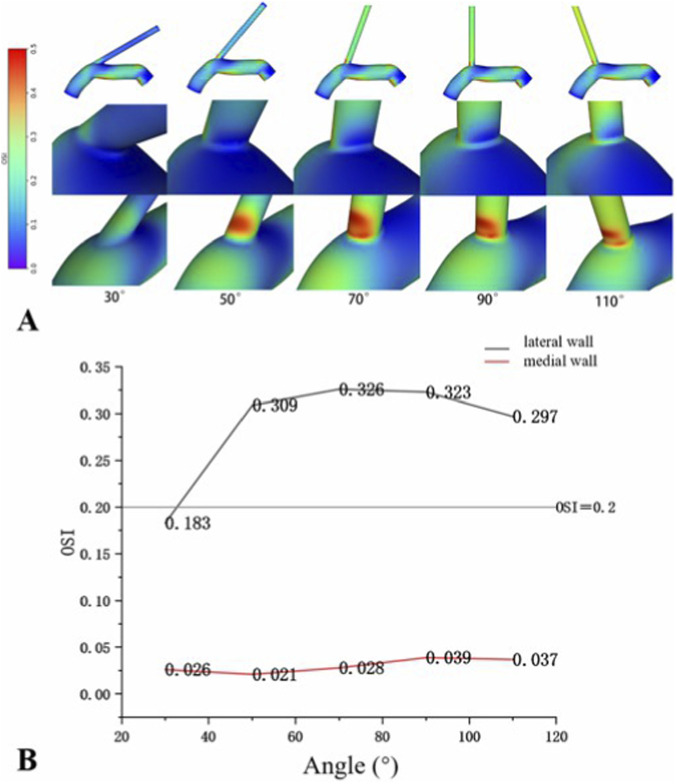
OSI distribution of VA-SCA based on the models. **(A)** The lateral wall consistently exhibited higher OSI values than the medial wall across all models. **(B)** OSI on lateral wall: all OSI >0.2 (atherogenic risk threshold), peaking at 70°. As the angle increases, the OSI _lateral_ initially rises and subsequently declines, attaining its peak at 70°.

**FIGURE 4:**
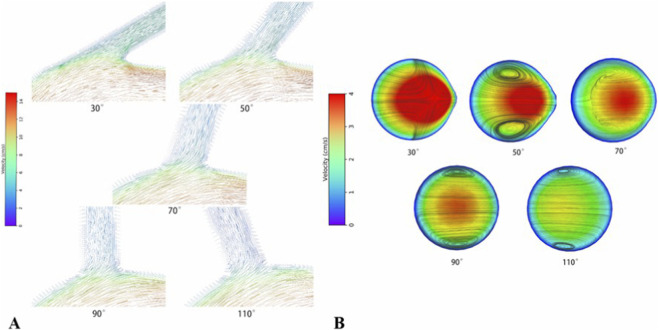
Visualisation of velocity distribution and location of the vortex in the VA-SCA bifurcation in 30°–110° simulated models. **(A)** No significant low-velocity flow zones were identified on the lateral wall of the VAO. **(B)** Coronal plane velocity streamlines illustrating the dynamic migration of the vortex core with increasing VA-SCA angle. With increasing VA-SCA angle, the vortex core relocated from the lateral wall (30°) to a more medial—yet still within the lateral portion—of the vessel at 50° and 70°, centralised at 90°, and ultimately shifted back to the vicinity of the lateral wall at 110°.

According to CFD models, both the medial and lateral walls at the VA-SCA bifurcation angles exhibited low TAWSS. Additionally, the lateral wall showed lower TAWSS values and a higher OSI in comparison to the medial wall across all angles (Figures 2A, 3A). The difference in flow velocity between the medial and lateral walls was not significant (Figure 4A).

### 3.4 The effect of angle on hemodynamics in the VA-SCA region

To investigate the effect of VA-SCA angle on hemodynamics, models with different angles were established and subjected to CFD analysis. Our research demonstrated that the angle affected TAWSS values. With an increase in the VA-SCA angle, TAWSS on the lateral wall initially decreased before subsequently increasing (Figures 2A,C). In contrast, the TAWSS on the medial wall exhibited an inverse pattern, first increasing and then decreasing (Figures 2A,B). More specifically, the lateral wall TAWSS peaked at 110° (1.404 dyn/cm^2^) and decreased to its lowest at 50° (1.167 dyn/cm^2^). Conversely, the medial wall TAWSS exhibited a maximal value of 50° (3.897 dyn/cm^2^) and subsequently decreased to a minimum value at 110° (3.375 dyn/cm^2^).

The OSI exhibited systematic variation in relation to the magnitude of the VA-SCA angle. The OSI value of the lateral wall initially increased and subsequently decreased with an increasing angle, reaching a peak at 70° (0.326) and remaining above 0.2, except at 30° (Figure 3B). The OSI value of the medial wall initially decreased and subsequently increased, attaining its minimum at 50° (0.021). Additionally, the high OSI zone on the lateral wall presented a dynamic change: it first expanded distally toward the VA, achieving its maximum area at 70°, and subsequently contracted proximally as the angle increased. Furthermore, the distribution of the high OSI zones was heterogeneous, with localised zones of moderately lower OSI exhibited within these at angles of 70°, 90°, and 110° (Figure 3A).

The VA-SCA angle also affected both flow velocity and flow patterns in the VA-SCA region. Our outcomes indicate that, at a consistent angle, flow velocities on the medial and lateral walls exhibited only minor differences. Furthermore, the angle slightly affected the velocity disparity between these walls within the same model (Figure 4A). However, the angle influenced the flow pattern by analysing the flow pattern at the VA-SCA junction surface (Figure 4B). We noticed that the vortexes were observed across all angles. With an increase in angle, the vortex centre migrated from the lateral wall to the medial wall. The vortex centre was positioned at the centre of the vessel at an angle of 90°. At an angle of 110°, the vortex centre relocated toward the outer wall. Furthermore, the vortex formation was most significant at a VA-SCA angle of 50°.

### 3.5 Validation by patients’ data

**FIGURE 5:**
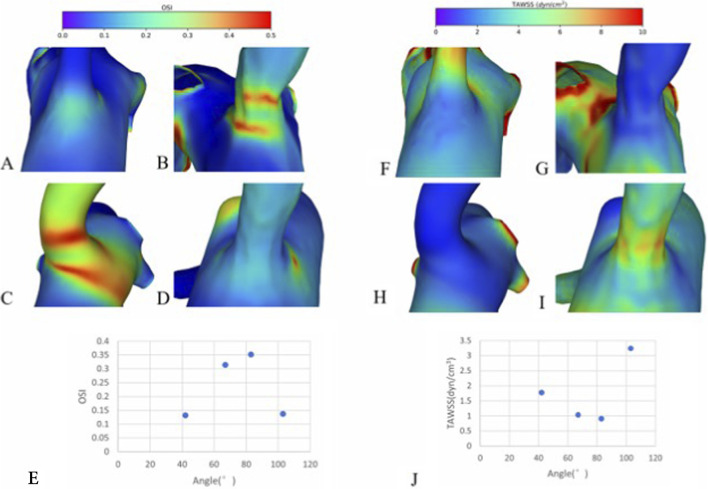
TAWSS and OSI on the lateral wall across different angles in patient-specific models. **(A–D)** Series of OSI distributions at representative VA-SCA angles of 42°, 67°, 83°, and 103°. **(E)** Corresponding trend of OSI variation with angle. **(F–I)** TAWSS distributions at the same set of VA-SCA angles (42°, 67°, 83°, 103°). **(J)** Associated trend of TAWSS variation with angle.

To validate the reliability of our simulation results, patient-specific models were reconstructed from CTA data for one representative case within each of four VA-SCA angle ranges: 30°–50°, 50°–70°, 70°–90°, and 90°–110°. The TAWSS and OSI were calculated for the lateral walls of the VAO in these models. The results confirmed that the hemodynamic trends observed in patient-specific models were consistent with those derived from the idealised simulations: as the VA-SCA angle increased, TAWSS exhibited an initial decrease followed by an increase, whereas OSI showed an initial increase followed by a decrease (Figure 5).

## 4 Discussion

Morphological factors may predict abnormal hemodynamics and potentially elevate the risk of AS. This study investigated the impact of VAO morphological factors on AS and explored potential mechanisms from a hemodynamic perspective. The first section of this research, through retrospective clinical data, identified VAO curvature and VA-SCA bifurcation angle as critical morphological parameters influencing the degree of VAO stenosis. The second part, based on clinical research, utilised CFD simulation to create idealised models of the left VA-SCA at various VA-SCA angles (30°, 50°, 70°, 90°, 110°). The results indicated that across all angles, the TAWSS of the lateral VA-SCA wall consistently remained lower than that of the medial wall. The OSI of the lateral wall consistently surpassed that of the medial wall, potentially correlating with the increased incidence of AS on the lateral VAO wall. As the angle increased, TAWSS first decreased and then increased, whereas OSI initially increased before decreasing. We considered that the angle of VA-SCA may affect the course of AS by influencing hemodynamics, and we hypothesised that VA-SCA angles ranging from 50° to 70° may be the critical angular range most predisposed to AS at the VAO, but these results were further verified with data from the real world.

Traditional etiological studies on AS have focused on systemic risk factors, including hypertension, hyperlipidemia, hyperhomocysteinemia, and diabetes (Chen H. et al., 2025). Nonetheless, conventional factors fail to fully elucidate the localised occurrence of AS and subsequent stenosis within the vessels. Recent developments in CFD have redirected research emphasis to the influence of local morphology in AS at VAO, where its morphological geometric features have been recognised to affect disease progression through modifications in hemodynamics. The change in hemodynamics was quantifiable by parameters such as TAWSS and OSI. Concurrently, these morphological features may function as potential imaging biomarkers for predicting the presence and progression of AS.

According to the retrospective clinical investigation section, severe tortuosity and larger VA-SCA angles constituted the independent risk factors for atherosclerosis at the VAO. In a previous study, the degree of tortuosity at the VAO influences the development of AS (Cagnie et al., 2006). However, no research has examined the correlation between VAO curvature and AS from a hemodynamic perspective. For the coronary artery, studies on the correlation between vascular tortuosity and AS remained controversial. Prior studies have demonstrated that elevated vascular tortuosity decreases distal perfusion pressure while increasing TAWSS at curved segments, consequently delaying the progression of AS, corroborated by CFD simulations (Vorobtsova et al., 2016). In contrast, Wong et al. (Wong et al., 2020) reported that highly tortuous coronary arteries induce turbulent flow circulation during blood transit, promoting AS and subsequently stenosis. Given these conflicting findings, the influence of VAO tortuosity on AS warrants additional elucidation via CFD modelling.

As evaluating VA-SCA tortuosity requires complex indices of three-dimensional curvature variations, limiting its clinical practicality, the VA-SCA angle offers a more intuitive and easily measurable alternative, positioning it as a promising imaging biomarker. In the second part of this study, therefore, we constructed idealised VA-SCA models with varying angles in order to systematically investigate the influence of angular variation on local hemodynamic parameters.

Hemodynamic characteristics shared exhibited commonalities across different VA-SCA angles. The lateral walls of the VAO exhibited low TAWSS (all <4 dyn/cm^2^) and high OSI regions (all >0.2) (Ekmejian et al., 2024). At the same angle, the TAWSS of the lateral wall was lower than that of the medial wall. Normal TAWSS values ranged from 10 to 70 dyn/cm^2^, with TAWSS <4 dyn/cm^2^ considered associated with AS. OSI values ranged from 0 to 0.5; OSI >0.2 significantly increased the risk of AS, while lower OSI values correlated with more stable blood flow (Cho et al., 2024). Recent studies have demonstrated that regions of low TAWSS often overlap with areas of high OSI, where atherosclerotic plaques of VAO are typically located (Ekmejian et al., 2024). Simonetto et al. (Simonetto et al., 2019) conducted an analysis of 91 coronary arteries, which revealed that 79% of atherosclerotic plaques at VA-SCA bifurcations were situated on the lateral wall, aligning with the regions of low TAWSS and high OSI identified in our study. Chen et al. (Chen Y. et al., 2025) identified high OSI zones on the lateral wall of the VA-SCA bifurcation, aligning with the results of this study. Katakia et al. (Katakia et al., 2022) previously demonstrated the relationship between the magnitude of the coronary bifurcation angle and AS through CFD simulations and molecular biology validation. It was shown that CFD simulations of coronary bifurcation geometries indicate the presence of regions with low WSS and high OSI on the side walls. 3D printing technology was utilized to fabricate geometric models with different bifurcation angles, and endothelial cells were cultured on the inner walls of each model under pulsatile blood flow conditions. Results indicated that areas characterized by low WSS and high OSI demonstrated overexpression of intercellular adhesion molecule-1 (ICAM-1) and vascular cell adhesion molecule-1 (VCAM-1), alongside increased macrophage adhesion. We proposed that low TAWSS and high OSI together influenced the hemodynamic properties of the VAO, making it this anatomical site susceptible to AS. Further investigations should be required to determine whether VAO shares analogous molecular biological mechanisms with coronary arteries.

In contrast to the findings of Chen et al. (Chen Y. et al., 2025), our investigation did not identify a distinct low-velocity flow zone on the lateral wall of the VAO when compared to the medial wall. This discrepancy may arise from variations in key parameters employed in the geometric models of the two studies. In the study by Chen et al. (Chen Y. et al., 2025), the SCA diameter of 9 mm was established, resulting in the emergence of a distinct low-velocity flow region on the lateral wall when the VA diameter was 5 mm. However, when the VA diameter measured 3 mm, the velocity difference between the medial and lateral walls was minimal, and no distinct low-velocity region was identified on the outer wall. The model parameters determined in this study (SCA diameter, 8.4 mm; VA diameter, 3.3 mm) were comparable to those of Chen’s model, which had a diameter of 3 mm, with an insignificant difference in flow velocity. From a hemodynamic perspective, significant differences in SCA and VA diameters resulted in minimal blood flow diversion from the SCA to the VA, characterized by a slow velocity, which leaded to minimal velocity differences between the inner and outer walls, resulting in indistinct low-velocity zones on the outer wall. In conclusion, we hypothesised that the VA-to-SCA diameter ratio might be one of the morphological factors affecting VAO hemodynamics and contributing to the development of AS. Meanwhile, we infered that whether SCA stenosis influenced the development of AS by altering the VAO flow field environment. But further research is necessary to determine this.

Moreover, this study observed vortex formation within the VAO across all VA-SCA angles. Previous studies have shown that abnormal blood flow patterns readily cause macrophages and platelets to accumulate in these vortex regions. Meanwhile, existing investigations have revealed that LDL distribution exhibits polarity, with increased LDL concentrations found in areas of abnormal flow, including secondary flows and vortices, in contrast to regions characterised by laminar flow (Xie et al., 2013). The vortex environment in this region may serve as a pathogenic factor facilitating the occurrence of AS.

The VA-SCA angle affected VAO hemodynamics. Our research identified a non-linear correlation between this angle and essential hemodynamic indicators (TAWSS, OSI). With an increase in the VA-SCA angle, the lateral wall TAWSS initially decreased before subsequently increasing, attaining its minimum at an angle of 50°. Conversely, the OSI of the lateral wall first rose and then declined, reaching its maximum at an angle of 70°. Our findings diverge from those of Chen et al. (Chen Y. et al., 2025), who exclusively reported elevated OSI and reduced TAWSS with increasing VA-SCA angle at the lateral wall of VAO. The discrepancy between studies may be attributed to the broader range of angles (30°–110°) utilised in our research, in contrast to the relatively limited range (30°, 45°, 60°) employed by Chen et al. (Chen Y. et al., 2025). The restricted range of angles may hinder a thorough understanding of the nature of TAWSS and OSI changes with angle variation. Our findings indicated that the VA-SCA angle between 50° and 70° may constitute a critical range most predisposed to the development of AS at the VAO. An abnormal hemodynamic environment, characterised by low TAWSS and high OSI within this angular range, may be the potential mechanism inducing the occurrence of AS. Clinical studies regarding the coronary arteries further substantiated our conclusions. Pinho et al. (2019) found comparable results in coronary arteries, indicating that the angle between the left anterior descending artery and the left circumflex artery had a negative correlation with WSS, suggesting that as the angle increased, WSS decreased, thereby elevating the risk of AS. Katakia et al. (2022) utilised CFD to show that an increase in the angle of the right coronary artery resulted in an initial decrease in WSS, followed by an increase, with the greatest susceptibility to stenosis observed at an angle of 60°.

The VA-SCA angle affected the vortex at the VAO centre. Our observation indicated that an increase in the VA-SCA angle resulted in dynamic migration of the vortex centre at the bifurcation, transitioning from a position favouring the outer wall to one nearer the inner wall, and subsequently returning towards the outer wall. Chen H. et al. (2025) found that the vortex centre consistently exhibited a bias towards the outer wall, which may be associated with the narrower model angle range employed in their study. Our research findings also considered that the most significant vortices occurred at a VA-SCA angle of 50°, aligning with the hypothesis mentioned above that angles between 50° and 70° were the most susceptible range for VAO to induce AS. The migration of vortex centres may be linked to the progression of AS; however, further research is necessary.

Furthermore, our study observed an elliptical, mildly low OSI region within the high OSI zone of the lateral wall at larger angles (70°, 90°, 110°). The region gradually transitioned towards the proximal VAO and diminished progressively with an increase in angle. Chen H. et al. (2025) also noted the comparable phenomenon that may be linked to the occurrence of AS.

Herein, we put forth a detailed account of CFD simulations; the outcomes of the study can assist clinicians in atherosclerosis risk-assessment prediction from 3D-angiograms. Specifically, this study indicates that VA-SCA angles within a specific range (notably 50°–70°) foster a hemodynamic environment favourable to AS at the VAO. Patients exhibiting angles inside this range may encounter an elevated clinical risk of AS and necessitate heightened scrutiny throughout diagnosis and treatment. Neurosurgeons should prioritise more active intervention strategies, like intensified secondary prevention and enhanced imaging surveillance, for cases with VA-SCA angles in this range, even though the current study only finds a general anatomical trend toward AS susceptibility rather than a definitive diagnostic threshold.

## 5 Conclusion

This study comprehensively examined the relationship between VAO morphology and AS, which integrated clinical retrospective analysis with CFD simulation. The primary conclusions were outlined below. Firstly, clinical retrospective studies indicated that a larger VA-SCA angle and severe VAO curvature were independent risk factors influencing AS at VAO. Secondly, CFD simulations and patient data both showed that an aberrant hemodynamic environment with low TAWSS and high OSI was present in the lateral wall of VAO. Increasing the VA-SCA angle, TAWSS initially decreased and then increased, whereas OSI inverted, which suggested that within a certain angular range, increasing the angle elevated the risk of AS.

## 6 Limitations and future work

Despite the fact that the study was the first to incorporate clinical retrospective analysis with CFD simulation for the examination of AS at the VAO, it has limitations.

Firstly, the clinical aspect of this investigation was founded on a single-centre retrospective design, which inevitably constrains the degree of evidence. Consequently, the results necessitate confirmation by extensive, multicenter prospective cohort investigations. Of note, diameter stenosis, while clinically familiar, does not serve as the gold standard for functional flow limitation. Furthermore, in the forthcoming research, we intend to integrate functional hemodynamic indicators (e.g., ultrasound-derived flow parameters, pressure wire-derived fractional flow reserve) to directly authenticate the correlation between morphological characteristics and actual hemodynamic importance.

Secondly, CFD simulations were based on idealised models obtained from the left VA-SCA geometry. However, the left and right hemispheres have different actual anatomical configurations and hemodynamic conditions, with the right hemisphere exhibiting a more complex structure. Therefore, additional validation is required to verify that the results of this study are applicable to right-sided VAO.

Thirdly, in the simulation, the vessel wall was considered a rigid structure, neglecting interactions between the blood and the wall. Fluid-structure interaction (FSI), which models the vessel wall as an elastic material, demonstrated higher sensitivity and accuracy in regions with low WSS than CFD, according to a study by Wang et al. (Wang et al., 2021; Moghadasi et al., 2024a; Moghadasi et al., 2024b). While this study’s rigid-wall assumption allowed for the detection of angle-dependent hemodynamic changes, it inevitably ignores vessel wall compliance and may induce biases. Future research will use FSI to investigate the hemodynamic connection between AS and VA-SCA angle in more physiologically relevant settings.

Ultimately, uniform boundary conditions were implemented throughout all simulations to isolate the impact of the VA-SCA angle. This assumption may influence the absolute hemodynamic values. Subsequent research will include sensitivity studies of boundary conditions to evaluate their impact on the outcomes.

## Data Availability

The original contributions presented in the study are included in the article/supplementary material, further inquiries can be directed to the corresponding authors.
